# A comparison of five methods to maximize RNA and DNA isolation yield from adipose tissue

**DOI:** 10.7717/peerj.17071

**Published:** 2024-05-03

**Authors:** Pawel Dabrowski, Marta Rasmus, Arkadiusz Jundzill, Tomasz Drewa, Marta Pokrywczynska

**Affiliations:** 1 Chair of Urology and Andrology, Department of Regenerative Medicine, Collegium Medicum, Nicolaus Copernicus University, Bydgoszcz, Poland; 2 Department of Plastic, Reconstructive and Aesthetic Surgery, Collegium Medicum, Nicolaus Copernicus University, Bydgoszcz, Poland

**Keywords:** RNA, DNA, Isolation, Nucleic acid, Adipose tissue, WAT

## Abstract

Adipose tissue in the human body occurs in various forms with different functions. It is an energy store, a complex endocrine organ, and a source of cells used in medicine. Many molecular analyses require the isolation of nucleic acids, which can cause some difficulties connected with the large amount of lipids in adipocytes. Ribonucleic acid isolation is particularly challenging due to its low stability and easy degradation by ribonucleases. The study aimed to compare and evaluate five RNA and DNA isolation methods from adipose tissue. The tested material was subcutaneous porcine adipose tissue subjected to different homogenization methods and RNA or DNA purification. A mortar and liquid nitrogen or ceramic beads were used for homogenization. The organic extraction (TriPure Reagent), spin columns with silica-membrane (RNeasy Mini Kit or High Pure PCR Template Preparation Kit), and the automatic MagNA Pure system were used for the purification. Five combinations were compared for RNA and DNA isolation. Obtained samples were evaluated for quantity and quality. The methods were compared in terms of yield (according to tissue mass), purity (A260/280 and A260/230), and nucleic acid degradation (RNA Integrity Number, RIN; DNA Integrity Number, DIN). The results were analyzed statistically. The average RNA yield was highest in method I, which used homogenization with ceramic beads and organic extraction. Low RNA concentration didn’t allow us to measure degradation for all samples in method III (homogenization with ceramic beads and spin-column purification). The highest RNA quality was achieved with method IV using homogenization in liquid nitrogen and spin column purification, which makes it the most effective for RNA isolation from adipose tissue. Required values of DNA yield, purity, and integrity were achieved only with spin column-based methods (III and IV). The most effective method for DNA isolation from adipose tissue is method III, using spin-columns without additional homogenization.

## Introduction

Adipose tissue in the human body occurs in various types with different morphology and functions. Among these tissues, the most known and the most common are white adipose tissue (WAT), primarily responsible for energy storage, and brown adipose tissue (BAT), involved in converting lipids into heat energy (Peirce, Carobbio & Vidal-Puig, 2014; Cypess et al., 2009). We can also distinguish beige/brite adipose tissue, which combines morphological features and functions of white and brown adipose tissues (Bartelt & Heeren, 2014), pink adipose tissue, which occurs mainly in women during pregnancy and lactation (Giordano et al., 2014) and marrow adipose tissue (MAT) or bone marrow adipose tissue (BMAT) which is a part of the bone marrow. Its function needs to be better understood (Cohen et al., 2012; Al-Nbaheen et al., 2013).

Over the past few decades, the overall view on the role of adipose tissue in the human body has changed. It is now known that adipose tissue contains numerous cell types, including mature cells (adipocytes, fibroblasts, smooth muscle cells, endothelial cells, blood cells), progenitor cells (preadipocytes, endothelial, vascular and hematopoietic progenitor cells) and stem cells (MSCs–mesenchymal stem cells, HSCs–hematopietic stem cells, pericytes, supra-adventitial cells) (Fraser et al., 2006). Adipose tissue is a complex endocrine organ that secretes hormones, including leptin, adiponectin, resistin, and other products (angiotensinogen, inflammatory cytokines, complement factors, steroids, and components of the coagulation/fibrinolytic pathway) involved in metabolic, neuroendocrine, immune, and cardiovascular regulation (Kershaw & Flier, 2004; Ouchi et al., 2011).

Balance between different types of adipocytes and the processes they run is closely related to maintaining energy homeostasis (Mulya & Kirwan, 2016). It is not new that the accumulation of adipose tissue observed in obesity is linked to the increasing risk of many metabolic (including type 2 diabetes) and inflammatory, hormonal, or cardiovascular diseases, which are a growing problem in modern society. However, there is still a lot to know about the mechanisms of these connections (Fain, 2006; Schrover et al., 2016).

Research on the proliferation and differentiation of preadipocytes and adipose-derived stem cells and understanding the interaction between different types of adipose tissue may provide new knowledge in the fight against many metabolic diseases (Lee, Mottillo & Granneman, 2014; Lou & Liu, 2016). It can also benefit cell-therapeutic treatment of neuronal and myocardial diseases or tissue regeneration (Madonna & De Caterina, 2010; Pokrywczynska et al., 2013; Frese, Dijkman & Hoerstrum, 2016; Ciuffi, Zonefrati & Brandi, 2017). To understand the mechanisms behind these unique properties, it is necessary to perform further research, including molecular analysis like sequencing, polymerase chain reaction, microarrays, and northern and southern blot for evaluation of differentiation potential, cellular senescence, and specific gene expression. Extraction and purification of nucleic acids is a crucial step of any molecular analysis. Any diagnostic or practical application requires a sufficient amount of high-quality genetic material. Therefore, an appropriate method for isolation of genetic material is of paramount importance (Chomczynski, 1993; Zuk et al., 2002).

High-quality nucleic acids are characterized by purity and degradation level. Protein contamination can be determined by measuring absorbance at 260 and 280 nm. The A260/280 ratio for high-purity RNA should be around 2.0, and for DNA, 1.7–2.0. A lower ratio may indicate the presence of protein or other contaminants with an absorbance close to 280 nm. The A260/230 ratio can be used as a secondary measure of nucleic acid purity. In this case, a ratio between 2.0 and 2.2 is considered pure for RNA and DNA. The lower ratio may indicate contamination by substances that absorb at 230 nm, *e.g*., phenol or guanidine (Glasel, 1995; Lucena-Aguilar et al., 2016). A260/280 and A260/230 ratios give information about the purity of the nucleic acid solution and the presence of protein or other contaminants. Still, they do not inform about the actual integrity of the nucleic acid particles. The level of RNA degradation is determined by the RNA integrity number (RIN), which estimates integrity using gel electrophoresis and analysis of the ratios of 28S to 18S ribosomal RNA. The RIN is presented as a numerical value from 1 to 10, where 10 presents intact RNA and one is a highly degraded material (Schroeder et al., 2006). A RIN above eight indicates high-quality RNA, between five and eight moderately degraded samples, and below five degraded samples. RNA samples with RIN values above five are usually recommended to use for quantification of gene expression by RT-qPCR. Also, a RIN lower than six can significantly affect the sequencing results (Fleige & Pfaffl, 2006; Pazzagli et al., 2013; Kukurba & Montgomery, 2015). The level of DNA degradation is determined by DNA integrity number (DIN), calculated from several features obtained from the electrophoretic trace and provides a number score from 1 to 10. A high DIN value indicates intact DNA, and a low DIN value indicates degraded DNA. Typically, a DIN value above eight indicates high-quality DNA. However, studies show that samples with DIN below seven are still sufficiently intact for whole exome library preparation and successful sequencing (Viercing et al., 2017; Hiramatsu et al., 2023).

There are several methods, many commercial reagents, and kits for nucleic acid extraction on the market. Many of them are modified to achieve better performance for particular types of nucleic acid or intended for specific sources and types of tissues. Among the most popular ways to extract nucleic acids from cells and tissues are those based on liquid-phase separations. These protocols are mainly an improvement of the single-step method of RNA extraction with an acid guanidinium thiocyanate-phenol-chloroform mixture developed by Chomczynski & Sacchi (1987). RNA, DNA, and protein are separated into organic and aqueous phases and purified by a series of precipitations (Chomczynski, 1993; Chomczynski & Sacchi, 1987, 2006). There are also simple and convenient column methods that bind nucleic acid to specific membranes made of silica gel or other materials. Nucleic acid bound to the membrane is treated with a series of washes and centrifuges to remove contaminants. More advanced, automated techniques, like MagNA Pure Systems, use magnetic glass particle technology to bind nucleic acids during the washing steps. Some protocols combine various techniques, *e.g*., organic extraction with spin column-based purification, for better results. These methods vary not only in their degree of technological sophistication, time consumption, and price but also in the quality and quantity of the obtained material (Lacinova et al., 2008; Tan & Yiap, 2009).

Isolation of nucleic acid from adipose tissue can be problematic because of the relatively small number of cells compared to other tissues (*e.g*., liver, testis, and ovary) per 1 mg of tissue, and a small number of cells can affect a small amount of nucleic acids in samples. Some other difficulties are connected with the large amount of lipids in adipocytes, which can impact tissue homogenization, phase separation, and purification. Fat particles can settle on the walls of the tubes and pipette tips and make the material challenging to collect. In some protocols, additional centrifugation helps form a removable fat layer on the sample’s surface (Lacinova et al., 2008; Pratt et al., 2013; Cirera, 2013). Ribonucleic acid isolation is particularly difficult due to low stability and easy degradation by ribonucleases. To protect RNA from degradation, the collected samples from cell culture or tissue can be stored at low temperatures in commercially available stabilizing solutions, which are aqueous reagents that preserve RNA in samples for an extended period at a wide range of storage conditions. It also helps to reduce the need for cryopreservation or immediate processing of tissue samples (Hemmrich et al., 2010).

In this study, two protocols of tissue homogenization and three different methods of DNA and total RNA purification have been compared: traditional organic extraction, solid-phase extraction (spin-column with silica membrane), and automatic MagNA Pure System. It gives five protocols for RNA isolation: ceramic beads + organic extraction (method I), liquid nitrogen + organic extraction (method II), ceramic beads + spin column (method III), liquid nitrogen + spin column (method IV), ceramic beads + automatic purification (method V) and 5 similar protocols for DNA isolation: ceramic beads + organic extraction (method I), liquid nitrogen + organic extraction (method II), no homogenization + spin column (method III), liquid nitrogen + spin column (method IV), ceramic beads + automatic purification (method V). This study aimed to compare five methods of RNA/DNA extraction to identify the technique yielding RNA/DNA of the best quality.

## Materials and Methods

### Adipose tissue samples

The tested material was subcutaneous porcine adipose tissue collected from six adult domestic pigs during planned economic slaughter in a local slaughterhouse. Tissue samples (*n* = 18; three samples per animal) were immediately stabilized in RNAlater solution (Qiagen GmbH, Germany) and stored at 4 °C until the isolation procedure.

### Homogenization of adipose tissue

Adipose tissue was fragmented into 25 mg pieces for RNA and 20, 25, or 100 mg for DNA isolation (according to the manufacturer’s recommendations for each method). Samples were disrupted and homogenized using two different systems. The first system, using ceramic beads technology, includes a transfer of tissue fragments into MagNA Lyser Green Beads tubes (Roche, Mannheim, Germany), the addition of required lysis buffer or TriPure Isolation Reagent (Roche, Mannheim, Germany), and disruption using MagNA Lyser Instrument (Roche, Mannheim, Germany) set on 6.500 rpm for 20 to 60 s in 20 s cycles. Samples were cooled in a MagNA Lyser Rotor Cooling Block between the cycles to avoid degradation by heat stress and centrifuged according to the manufacturer’s protocol. In the second method, tissue samples were transferred into a mortar, frozen with liquid nitrogen, and disrupted. Then, the required reagent (lysis buffer or TriPure) was added to the disrupted sample, transferred into 1.5 ml nuclease-free centrifuge tubes, and processed according to the manufacturer’s protocol.

### Total RNA extraction

Samples after homogenization were subjected to three different methods of RNA isolation, including manual organic extraction with TriPure Isolation Reagent (Roche, Mannheim, Germany), silica membrane-based spin column technology using RNeasy Mini Kit (Qiagen, Hilden, Germany), and automated magnetic separation with MagNA Pure LC 2.0 Instrument (Roche, Mannheim, Germany). For extraction with TriPure Isolation Reagent, the supernatant obtained during homogenization was transferred to a fresh centrifuge tube, phase separation with chloroform was performed, RNA was precipitated from the colorless aqueous phase, washed with ethanol and resuspended in 30 μl of RNase-free water. For isolation with RNeasy Mini Kit, the supernatant obtained during homogenization was transferred to a fresh centrifuge tube, an equal volume of 70% ethanol was added, a sample was transferred to the RNeasy spin column, processed according to the manufacturer’s protocol and eluted with 50 μl of RNase-free water. For isolation with MagNA Pure LC RNA Isolation Kit III Tissue (Roche, Mannheim, Germany), 350 μl of the lysate was transferred into the Sample Cartridge, loaded into MagNA Pure LC 2.0 Instrument (Roche, Mannheim, Germany) with all necessary reagents and disposables, and performed automatically using preset protocol ‘RNA Tissue Fresh_frozen’ dedicated to the isolation of RNA from tissue. Combinations of these techniques with two methods of tissue homogenization give five protocols for RNA isolation:

- method I–homogenization with ceramic beads + organic extraction
- method II–homogenization in liquid nitrogen + organic extraction,
- method III–homogenization with ceramic beads + spin column purification,
- method IV–homogenization in liquid nitrogen + spin column purification,
- method V–homogenization with ceramic beads + automatic purification.

The protocols for RNA isolation are summarized in Table 1. The RNA samples were subjected to quantitative and qualitative analysis, including total RNA yield according to tissue mass, purity, and integrity.

**Table 1:** Summarized RNA isolation protocols.

Method	I	II	III	IV	V
**Amount of tissue (mg)**	~25	~25	~25	~25	~20
**Homogeni-** **zation**	Ceramic beads	Liquid nitrogen, mortar and pestle	Ceramic beads	Liquid nitrogen, mortar and pestle	Ceramic beads
**Purification**	Organic extraction (TriPure Isolation Reagent)	Organic extraction (TriPure Isolation Reagent)	Spin column with silica-membrane (RNeasy Mini Kit)	Spin column with silica-membrane (RNeasy Mini Kit)	Magnetic glass beads (MagNA Pure LC RNA Isolation Kit III)
**Elution**	RNase-free water	RNase-free water	RNase-free water	RNase-free water	Elution buffer

### Total DNA extraction

Samples after homogenization were subjected to three different methods of DNA isolation, including manual organic extraction with TriPure Isolation Reagent (Roche, Mannheim, Germany), spin-column technology using High Pure PCR Template Preparation Kit (Roche, Mannheim, Germany), and automated magnetic separation with MagNA Pure LC 2.0 Instrument (Roche, Mannheim, Germany). For extraction with TriPure Isolation Reagent (Roche, Mannheim, Germany), the supernatant obtained during homogenization with ceramic beads or liquid nitrogen was transferred to a fresh centrifuge tube, phase separation with chloroform was performed, DNA was precipitated from the interphase and red organic phase, washed with sodium citrate, ethanol and dissolved in 100 μl of 8 mM NaOH. Protocol for DNA isolation with a High Pure PCR Template Preparation Kit (Roche, Mannheim, Germany) does not include mechanical homogenization (it is optional), which is why one group of tissue samples was digested with proteinase K and another was homogenized with liquid nitrogen, mortar, and pestle before digestion and then processed according to manufacturer’s guide. For isolation with MagNA Pure LC DNA Isolation Kit II Tissue (Roche, Mannheim, Germany), 80 μl of the lysate was transferred into the Sample Cartridge, loaded into MagNA Pure LC 2.0 Instrument (Roche, Mannheim, Germany) with all necessary reagents and disposables, ‘DNA II Tissue’ protocol was chosen, and all further steps were performed automatically. Combinations of these techniques with two methods of tissue homogenization give five protocols for DNA isolation:

- method I–homogenization with ceramic beads + organic extraction,
- method II–homogenization in liquid nitrogen + organic extraction,
- method III–no homogenization + spin column purification,
- method IV–homogenization in liquid nitrogen + spin column purification,
- method V–homogenization with ceramic beads + automatic purification.

The protocols for DNA isolation are summarized in Table 2. The obtained DNA samples were subjected to qualitative and quantitative analysis, including DNA yield according to tissue mass, purity, and integrity.

**Table 2:** Summarized DNA isolation protocols.

Method	I	II	III	IV	V
**Amount of tissue (mg)**	~100	~100	~25	~25	~20
**Homogeni-zation**	Ceramic beads	Liquid nitrogen, mortar and pestle	No homogenization (digestion with Proteinase K)	Liquid nitrogen, mortar and pestle (+digestion with Proteinase K)	Ceramic beads
**Purification**	Organic extraction (TriPure Isolation Reagent)	Organic extraction (TriPure Isolation Reagent)	Spin column with glass fiber fleece (High Pure PCR Template Preparation Kit)	Spin column with glass fiber fleece (High Pure PCR Template Preparation Kit)	Magnetic glass beads (MagNA Pure LC DNA Isolation Kit II)
**Elution**	8 mM NaOH	8 mM NaOH	Elution buffer	Elution buffer	Elution buffer

### Qualitative and quantitative analysis

The nucleic acid’s quantity and A260/280 nm absorbance ratio were analyzed with a NanoDrop Lite Spectrophotometer (Thermo Scientific, Waltham, Massachusetts USA). The A260/230 ratio was obtained using a Varioskan LUX microplate reader with a dedicated µDrop plate (Thermo Scientific, Waltham, Massachusetts, USA). RNA integrity was checked using a 2100 Bioanalyzer Instrument (Agilent, Santa Clara, California, USA) and a dedicated RNA 600 Kit (Agilent, Santa Clara, California, USA). TapeStation 4150 System with Genomic DNA ScreenTape Assay (Agilent, Santa Clara, California, USA) was used to analyze DNA integrity. Analyses were processed according to the manufacturer’s guides.

### Statistical analysis

Statistical analysis was performed using GraphPad Prism (GraphPad Software, La Jolla, California, USA). Data were assessed for normality (Shapiro-Wilk test) and subjected to appropriate parametric (ordinary one-way ANOVA) or non-parametric tests (Kruskal-Wallis test) with multiple comparisons. According to the statistical tests, data are presented as mean ± SD or median with IQR. Values of *p* < 0.05 were considered statistically significant.

## Results

### RNA yield, purity, and integrity

**Figure 1:**
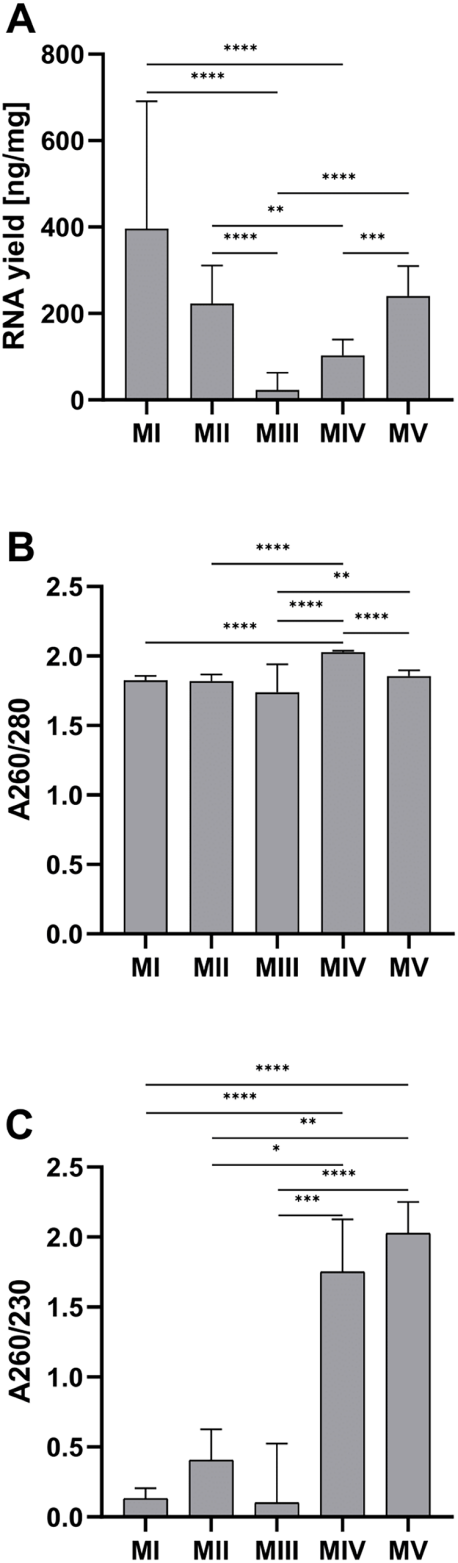
Comparison of RNA yield and purity for methods I-V. (A) Total RNA amount per 1 mg of tissue sample for methods I–V (data presented as median and IQR, *n* = 18, **p < 0.01, ***p < 0.0011, ****p < 0.0001); (B) RNA purity–A260/280 ratio for methods I–V (data presented as mean ± SD, *n* = 18, **p < 0.0037, ****p < 0.0001); (C) RNA purity–A260/230 ratio for methods I–V (data presented as median and IQR, *n* = 18, *p < 0.0408, **p < 0.0012, *** *p* < 0.0004, ****p < 0.0001).

The average RNA yield per tissue amount was the highest for homogenization with ceramic beads and organic extraction (method I)–281.80 (212.30–460.50) ng/mg and the lowest for ceramic beads homogenization with spin column purification (method III)–2.24 (1.55–25.75) ng/mg. There was a large discrepancy in the results for method III. Part of the samples was in the range of 69.2–117.4 ng/mg (*n* = 4), and the rest was in the range of 1.0–1.2 ng/mg (*n* = 14) (Fig. 1A). The homogenization in liquid nitrogen and spin column purification (method IV) with the result at 2.03 ± 0.01 for A260/280 and 1.75 (0.98–2.13) for A260/280 is the most effective. The lowest values of the A260/280 (1.74 ± 0.20) and A260/280 (0.10 (0.00–0.53)) were obtained in method III. In other methods, the A260/280 values exceed 1.8. Method IV and V (ceramic beads homogenization and automatic purification) are the only methods with acceptable A260/230 ratios, with 1.75 (0.98–2.13) and 2.01 (1.85–2.25), respectively (Figs. 1B and 1C).

**Figure 2:**
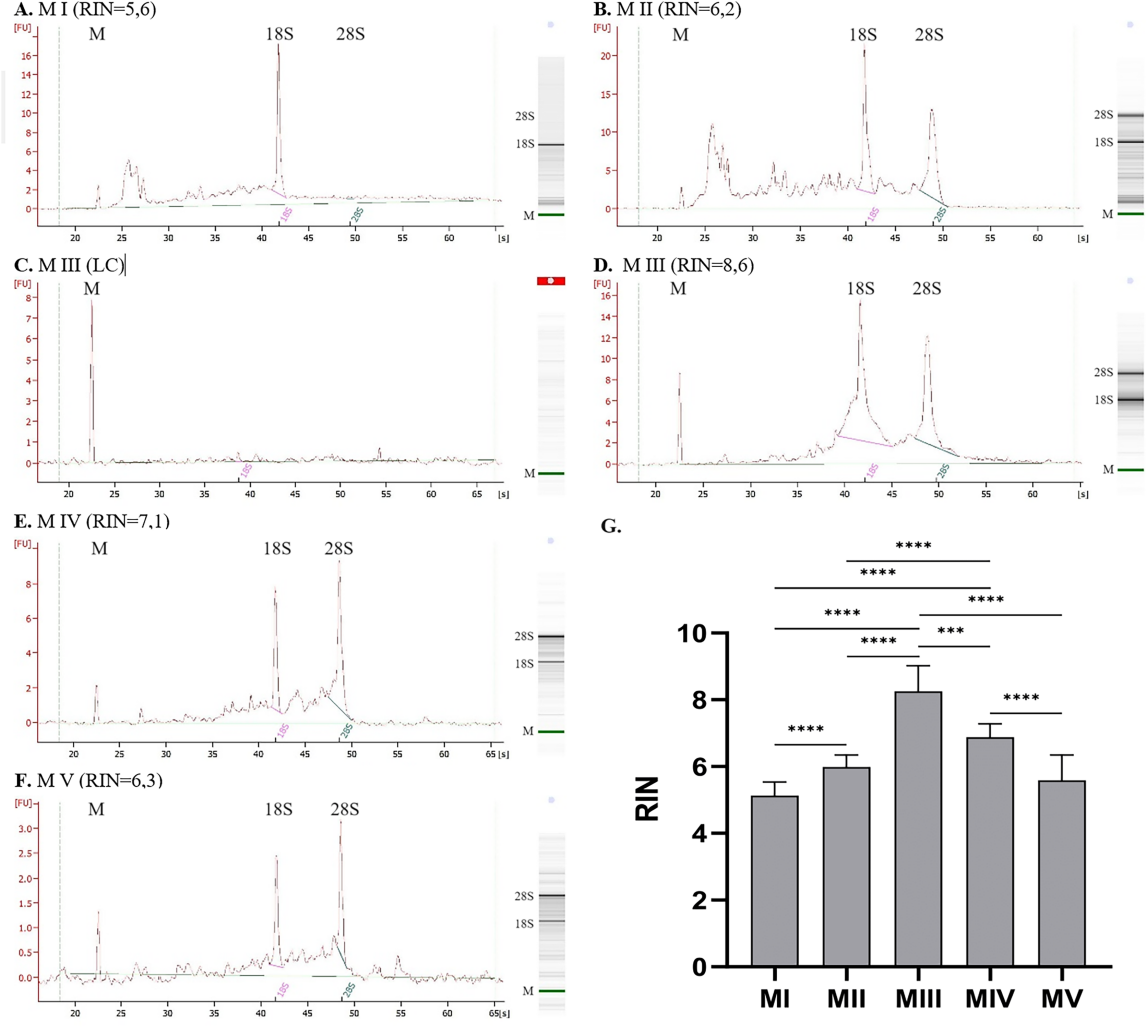
RNA integrity for methods I–V measured with 2100 Bioanalyzer instrument (exemplary electropherograms and gel-like images; M-marker, 28S–28S ribosomal RNA, 18S–18S ribosomal RNA). (A) RNA integrity for method I, (B) RNA integrity for method II, (C) RNA integrity for method III–sample with low RNA concentration, (D) RNA integrity for method III–sample with sufficient RNA concentration, (E) RNA integrity for method IV, (F) RNA integrity for method V; (G) Comparison of RNA Integrity Number (RIN) for methods I–V; (data presented as mean ± SD; *n* = 17 for method I and IV, *n* = 18 for method II and V, *n* = 4 for method III; ***p = 0.0001, ****p < 0.0001).

The quality of RNA can be evaluated based on the value of RIN (Fig. 2G). The highest RIN value (8.25 ± 0.77) was obtained for method III (ceramic beads homogenization with spin column purification) but only for four samples with sufficient RNA concentration. Measurements were not possible for the other 14 samples because the RNA concentration was too low (LC). The method with acceptable RIN values (6.88 ± 0.41) was method IV, combining homogenization in liquid nitrogen and spin column purification. The average RIN values are below 6 for the rest of the methods.

Figures 2A–2F presents results for RNA integrity measured with 2100 Bioanalyzer Instrument. The first peak on each electropherogram labeled as ‘M’ represents the marker; the following peaks correspond to 18S and 28S RNA. Graph for method I (homogenization with ceramic beads and organic extraction) presents a significant peak for 18S RNA and a lack of 28S subunits. There are also some smaller particles of RNA, which is noticeable for method II (homogenization in liquid nitrogen and organic extraction). It can indicate partially degraded material. Despite the similar RIN values for methods I and II (Figs. 2A and 2B), electropherograms and gel-like images differ. In method I, no peak and band correspond to 28S RNA, which is visible but small for method II. Additional elevation at the beginning of the graph may indicate sample contamination or the presence of 5S and 5.8S RNA. Results for method III are presented in Figs. 2C and 2D, where C is a sample with RNA concentration too low to measure the RIN parameter, and D is a sample with sufficient concentration–peaks for 28S and 18S subunits are clear. Electropherograms for method IV, combining homogenization in liquid nitrogen and spin column purification (Fig. 2E), and method V, combining ceramic beads homogenization and automatic purification (Fig. 2F), show distinct peaks for 28S and 18S ribosomal RNA with just a little smear between them.

The average values of measured parameters for all tested RNA isolation methods are summarized in Table 3.

**Table 3:** The average values of the A260/280 and A260/230 ratios, yield, and RIN for tested RNA isolation methods (^A^ – data presented as median and IQR; ^B^ – data presented as mean ± SD).

Method	Yield (ng/mg)^A^	A260/280^B^	A260/230^A^	RIN^B^
**MI**	281.80 (212.30–460.50)	1.83 ± 0.03	0.13 (0.05–0.21)	5.13 ± 0.41
**MII**	200.70 (148.6–288.90)	1.82 ± 0.05	0.41 (0.29–0.63)	5.98 ± 0.36
**MIII**	2.24 (1.55–25.75)	1.74 ± 0.20	0.10 (0.00–0.53)	8.25 ± 0.77
**MIV**	100.00 (80.05–135.10)	2.03 ± 0.01	1.75 (0.98–2.13)	6.88 ± 0.40
**MV**	222.70 (189.70–302.40)	1.86 ± 0.04	2.01 (1.85–2.25)	5.59 ± 0.76

### DNA yield, purity, and integrity

**Figure 3:**
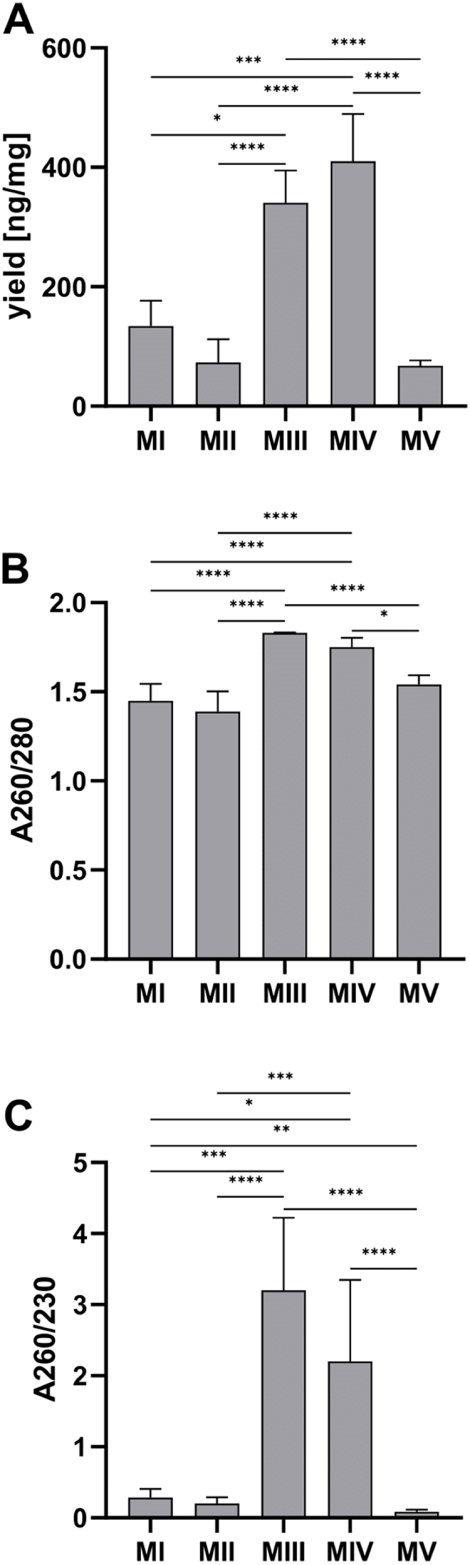
Comparison of DNA yield and purity for methods I–V. (A) Total DNA amount per 1 mg of tissue sample for methods I–V (data presented as median and IQR, *n* = 18, * *p* < 0.0125, *** *p* < 0.0002, **** *p* < 0.0001); (B) DNA purity–A260/280 ratio for methods I–V (data presented as median and IQR, *n* = 18, *p < 0.0118, ****p < 0.0001); (C) DNA purity–A260/230 ratio for methods I–V (data presented as median and IQR, *n* = 18, *p < 0.0362, **p < 0.0069, ***p < 0.0003, ****p < 0.0001).

Among tested methods, spin-column purification (methods III and IV) stands out with significantly higher DNA yield and purity than the other methods. The average DNA yield per tissue amount was the highest for method IV, combining homogenization in liquid nitrogen and spin column purification–410.20 (352.10–489.00) ng/mg and the lowest for the automatic method V (ceramic beads homogenization and automated purification)–67.65 (56.25–76.76) ng/mg (Fig. 3A). A260/280 and A260/230 ratios have the highest values for spin-column methods, respectively 1.83 (1.80–1.83) and 3.20 (3.00–7.10) for method III (spin column purification with no additional homogenization), 1.75 (1.73–1.80) and 2.20 (2.00–3.35) for method IV (homogenization in liquid nitrogen and spin column purification). There is no significant difference between the results for these methods. The purity of the rest of the methods was under recommended levels (Figs. 3B and 3C).

**Figure 4:**
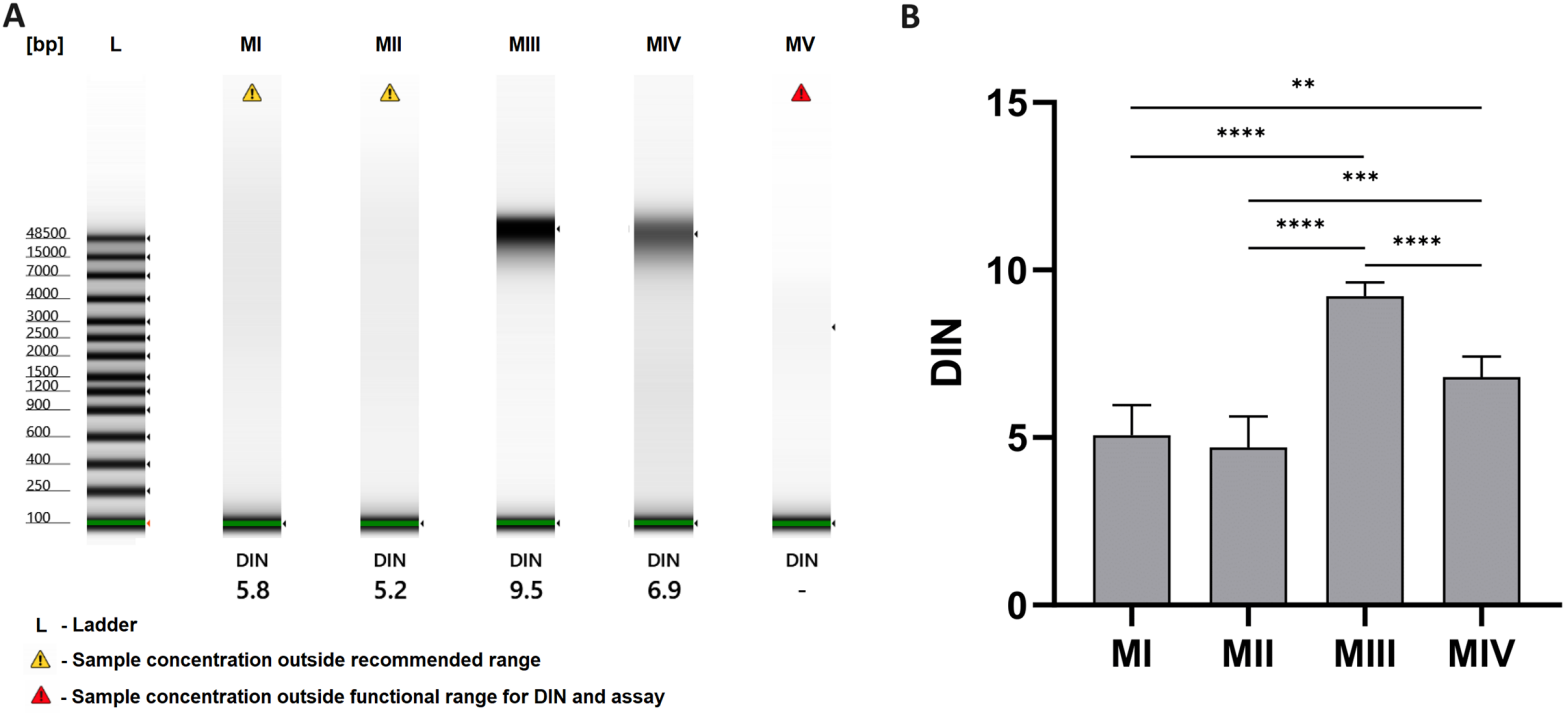
DNA integrity for methods I–V. (A) DNA integrity measured with 4150 TapeStation system (exemplary gel-like images); (B) comparison of DNA integrity number (DIN) for methods I–V. Data presented as mean ± SD, *n* = 6, **p < 0.0034, ***p < 0.0005, ****p < 0.0001.

The results of analysis with the TapeStation system allowed the evaluation of the integrity of DNA samples, which is presented as gel-like images in Fig. 4A and as a comparison of DNA integrity number (DIN) for methods I-V in Fig. 4B. Least degraded DNA samples were achieved with spin-column methods, especially method III, with an average DIN value of 9.22 ± 0.42 and a clear stripe of genomic DNA on a gel-like image. Additional homogenization in liquid nitrogen in method IV may cause higher degradation (blurred stripe on gel-like image) and lower DIN values–6.80 ± 0.61. DNA isolated with organic extraction (methods I and II) is characterized by DIN values lower than 6 and presented as a faded smear on the electropherogram. In method V (automatic purification), the sample concentration was too low to determine DIN.

The average values of measured parameters for all tested DNA isolation methods are summarized in Table 4.

**Table 4:** The average values of the A260/280 and A260/230 ratios, yield, and DIN for tested DNA isolation methods (^A^ – data presented as median and IQR; ^B^ – data presented as mean ± SD).

Method	Yield (ng/mg)^A^	A260/280^A^	A260/230^A^	DIN^B^
**MI**	134.00 (105.00–176.60)	1.45 (1.34–1.55)	0.29 (0.22–0.41)	5.07 ± 0.90
**MII**	73.44 (38.76–112.10)	1.39 (1.35–1.50)	0.20 (0.17–0.29)	4.70 ± 0.93
**MIII**	340.70 (284.60–394.70)	1.83 (1.80–1.83)	3.20 (3.00–7.10)	9.22 ± 0.42
**MIV**	410.20 (352.10–489.00)	1.75 (1.73–1.80)	2.20 (2.00–3.35)	6.80 ± 0.61
**MV**	67.65 (56.25–76.76)	1.54 (1.49–1.59)	0.09 (0.04–0.12)	–

## Discussion

Highlighted by many authors, the high content of lipids and relatively low number of cells in adipose tissue may cause difficulties in obtaining a large amount of high-quality nucleic acids. Comparing average RNA yield from various human and animal tissues, it can be stated that adipose tissue has a relatively low amount of RNA and DNA. Obtained values for RNA range from 20 to 280 ng/mg for different methods in this study are similar to those from skin, heart, or prostate and significantly lower than tissues like muscle, liver, or pancreas. The average DNA yield ranges from 70 to 428 ng/mg for methods compared in this study, which makes it several times less than the yield obtained from muscle, liver, lung, or thymus (Walker et al., 2016; Liska & Ruprecht, 1999; Biase et al., 2002; Ginzberg, Kafri & Kirschner, 2015; Stroh et al., 2021). Broad ranges of nucleic acid yield are mainly a result of the capabilities of the techniques used. Also, some differences may be due to individual animal characteristics and subcutaneous adipose tissue collection location.

Comparing the final RNA purity values obtained for methods in our study with the research carried out by Cirera (2013) and Lacinova et al. (2008), it can be stated that the spin column-based methods give RNA of the highest purity. Method IV using RNeasy Mini Kit and homogenization in liquid nitrogen gives even better results than spin-column methods used by Lacinova et al. (2008): the RNeasy Protect Mini Kit and dedicated for fatty tissues RNeasy Lipid Tissue Kit. Total RNA amount 100.00 (80.05–135.10) ng/mg and 2.03 ± 0.01 A260/280 ratio for method IV are significantly higher than 27.3 ± 15.1 ng/mg and 1.94 ± 0.16 for RNeasy Protect Mini Kit or 45.8 ± 19.4 ng/mg and 1.70 ± 0.10 for the RNeasy Lipid Tissue Kit (Lacinova et al., 2008).

The organic extraction (methods I and II) usually shows problems with the purity of the isolated nucleic acids, especially the A260/230 ratio, which is consistently very low–well below 1, for RNA and DNA isolation. It can be caused by remaining solvents (*e.g*., phenol and chloroform) or chaotropic salts (guanidine isothiocyanate) in the nucleic acid solution, and it can limit downstream applications. The A260/280 ratio for RNA in organic extraction methods is also significantly lower-around 1.8.

The organic RNA extraction protocol (using TRIzol reagent) developed by Roy et al. (2020) can help obtain significantly better RNA quality and yield than the manufacturer’s protocol. Modifying the TRIzol-to-tissue ratio from 10:1 to 1:2 and increasing tissue amount from 50–100 to 500 mg provides A260/230 values above 1.8. This protocol modification could also positively impact the results for organic RNA extraction using other reagents, such as TriPure in methods I and II.

The protocol for RNA isolation developed by Cirera (2013), which combines organic extraction (TRI Reagent) and spin column-based RNA purification (miRNeasy kit), gives significantly higher RNA purity than each of these methods separately: A260/280 ratio of 2.00 ± 0.049 (1.81 ± 0.023 for organic extraction and 2.01 ± 0.03 for spin column) and A260/230 ratio of 1.73 ± 0.189 (0.61 ± 0.25 for organic extraction and 1.43 ± 0.25 for spin column). Method IV (homogenization in liquid nitrogen and spin-column purification with RNeasy Mini kit) with an A260/280 ratio of 2.03 ± 0.01 and an A260/230 ratio of 1.75 (0.98–2.13) gives results comparable to the protocol developed by Cirera (2013).

In method V, which uses the automatic MagNA Pure system, it was possible to obtain a significantly higher yield (222.70 (189.70–302.40) ng/mg) and A260/280 ratio (1.86 ± 0.04) than in the similar method used by Lacinova et al. (2008) (33.7 ± 5.1 ng/mg, and 1.74 ± 0.04).

Comparison of methods I with II (organic extraction) and III with IV (spin-column purification) allows us to compare the effect of the selected homogenization method using the same RNA purification protocol. The type of homogenization had no significant impact on the results for methods I and II. However, the difference is significant for spin-column methods III and IV. For some samples in method III, the concentration was too low, making it impossible to measure the RIN value. The low concentration also had an impact on the average purity values and obtained amount of RNA. For this method, the A260/280 ratio with the value of 1.74 ± 0.20 placed below the recommended range but still seems acceptable for some purposes. However, the ratio cannot be accurately calculated for samples close to the lower detection limits of the NanoDrop Lite Spectrophotometer, and the concentration of most samples is close to 0. The average value is also overestimated by samples with higher RNA concentrations, whose purity is adequate. The low concentration of RNA may be due to the membrane clogging by the remaining fat in the mixture. Such results testify to the lack of repeatability of method III and exclude it as an effective method of RNA isolation. Combining the column method with homogenization using a mortar and liquid nitrogen in method IV brought much better results–RNA with higher purity and yield was obtained. It follows that the homogenization in liquid nitrogen may eliminate the problem of column clogging during RNA isolation. It could be affected by the degree of tissue fragmentation and lipids released from the cells. Homogenization with a mortar and liquid nitrogen provided acceptable quality of RNA, as evidenced by clear peaks and bands corresponding to 18S and 28S RNA and RIN values around seven, which is suitable for further molecular analysis. In method III, using ceramic beads homogenization, very low concentrations were obtained in most tests (*n* = 14), which made it impossible to visualize the degree of RNA degradation. For samples with higher concentrations (*n* = 4), the obtained material is characterized by a low degradation (RIN values above 8).

Among tested methods, spin-column purification (methods III and IV) stands out with significantly higher DNA yield, A260/230 and A260/280 ratio than the others. Desired values have been achieved only for these methods. Comparison of the results for method I with II and method III with IV can help to notice that different way of homogenization has no significant impact on the yield and purity of DNA samples. Additional homogenization with liquid nitrogen, mortar, and pestle in method IV has no significant effect on differences in purity and yield of the resulting material. This only helps to shorten the digestion time of the tissue from about an hour to 10 min. The least degraded DNA samples were also achieved with spin-column methods, although additional homogenization in liquid nitrogen (method IV) may cause higher degradation. Method III gives a higher DNA yield (340.70 (284.60–394.70) ng/mg) than the spin-column method tested by Stroh et al. (2021), which used homogenization with a disposable plastic pestle and DNeasy Blood & Tissue kit (52 ± 14 ng/mg.). The A260/280 ratio for method III is 1.83 (1.80–1.83), which is exactly in the desired range when A260/280 for the technique used by Stroh et al. (2021) is slightly above (2.4 ± 0.2).

DNA isolated with organic extraction is characterized by low integrity with DIN values below six. Magnetic separation (method V) allows us to obtain a meager DNA yield compared to other methods, and it may be due to the fact that part of the homogenized tissue with the lysis buffer (buffer used in this method in a small amount) is stuck in between ceramic beads. Despite high technological advancement, the automated RNA isolation method used in the study doesn’t give better results in quantity, purity, and quality of RNA than other methods. However, it is not efficient enough for the isolation of DNA from adipose tissue.

In addition to the desired yield, purity, and quality of the resulting material, selecting the appropriate tissue homogenization and nucleic acid isolation method depends on many other factors. Financial aspects, time availability, and the number of tested samples can be distinguished here. The advantages and disadvantages of the nucleic acid purification methods used are listed in Table 5.

**Table 5:** Advantages and disadvantages of the nucleic acid isolation techniques.

	Organic extraction	Spin column purification	Automated purification with magnetic beads
**Advantages**	- Requires basic lab equipment - RNA, DNA, and proteins can be isolated from the same sample - Low cost	- Requires basic lab equipment - Ready-to-use kit format - Short execution time	- Processing multiple samples at once in a short time - No use of hazardous chemical compounds
**Disadvantages**	- Time-consuming (especially when working with many samples) - Requires high precision (risk of contamination of the final sample with other types of nucleic acids or proteins) - Uses hazardous chemical compounds	- Some sample types or incomplete homogenization can clog the membranę - Working with multiple samples can increase time consumption	- Requires specialized equipment - Viscous samples can impair the migration of magnetic beads - High cost

## Conclusion

In conclusion, methods I, II, IV, and V were effective for RNA isolation. Because of the lack of repeatability, method III is not a suitable method for RNA isolation from adipose tissue. The highest RNA purity and integrity with appropriate yield was achieved with method IV using homogenization with liquid nitrogen and spin-column purification. Required values of DNA yield, purity, and integrity were achieved only with spin column-based methods (III and IV). The most effective method for DNA isolation from adipose tissue is method III, which uses spin-columns without additional homogenization, characterized by high DNA yield, purity, and integrity. Extraction methods I and II and automatic method V are not efficient for DNA isolation from adipose tissue because of low DNA purity and integrity below acceptable levels. These two selected methods provide an appropriate amount of RNA and DNA from adipose tissue of a quality suitable for further analysis using methods such as PCR, sequencing, and microarrays.

## Supplemental Information

10.7717/peerj.17071/supp-1 Supplemental Information 1 Raw data for RNA isolation with methods I-V.

10.7717/peerj.17071/supp-2 Supplemental Information 2 Raw data for DNA isolation with methods I-V.

10.7717/peerj.17071/supp-3 Supplemental Information 3 Statistical analysis for RNA isolation with methods I-V.

10.7717/peerj.17071/supp-4 Supplemental Information 4 Statistical analysis for DNA isolation with methods I-V.

## Additional Information and Declarations

### Data Availability

The following information was supplied regarding data availability: The raw data is available in the Supplemental Files.
